# Comparison of the health-related quality of life, CD4 count and viral load of AIDS patients and people with HIV who have been on treatment for 12 months in rural South Africa

**DOI:** 10.1080/17290376.2013.807070

**Published:** 2013-06-18

**Authors:** Jude Igumbor, Aimee Stewart, William Holzemer

**Keywords:** AIDS/HIV, CD4 cell count, health-related quality of life, viral load, WHOQOL-HIV, VIH/SIDA, le nombre de cellules CD4, la qualité de vie liée à la santé, de la charge virale

## Abstract

This study compared the level of CD4 count, viral load and health-related quality of life (HRQOL) between treatment-naïve AIDS patients and a cohort of people living with HIV who have been on treatment for 12 months. This study is based on a secondary data analysis of the records of 642 people with HIV consisting of 311 treatment-naïve AIDS patients and 331 people with HIV who have been on treatment for 12 months. The study findings are mostly presented in tables and analysed using the *t*-test to compare HRQOL scores, CD4 count and viral load in the two groups. The study generally noted poor financial capacity and low activity tolerance among the participants. Significant changes were noted in all the domains of HRQOL compared between the treatment-naïve patients and the 12 months treatment cohort. In the same manner, the median CD4 cell count and viral load differed significantly between both groups. The treatment-naïve and the 12 months treatment cohorts consistently reported much lower quality of life scores in the level of dependence domain which includes the measures of mobility, activity of daily living, dependence on medication and work capacity. There were little or no associations between the biomedical markers (CD4 count and viral load) and HRQOL indicators. However, the quality of life tended to increase with increase in the CD4 cell count. The poor to no association between the biomedical markers and HRQOL indicators show that these cannot be direct proxies of each other and that the CD4 cell count and viral load alone may be inadequate eligibility criteria for social support.

## Abstract (FR)

La présente étude est une comparaison du niveau de CD4, de la charge virale et de la qualité de vie liée à la santé entre les patients n'ayant jamais reçu de traitement de SIDA et une cohorte de personnes vivant avec le VIH traitées pendant 12 mois. Elle est basée sur des données secondaires des dossiers de 642 personnes composée de 311 patients n'ayant jamais reçu de traitement de SIDA et 331 personnes vivant avec le VIH traitées pendant 12 mois. Nous avons utilisé pour cette étude le test T pour comparer les résultats de la qualité de vie liée à la santé, le nombre de CD4 et la charge virale dans les deux groupes. Au cours de l'étude l'on a noté en général une faible capacité financière et une faible tolérance d'activité parmi les participants. Des changements importants ont été observés dans tous les domaines de la qualité de vie liée à la santé entre les patients n'ayant jamais reçu de traitement et la cohorte de douze mois de traitement. De même, la numération médiane des cellules CD4 et la charge virale présentaient une différence importante entre les deux groupes. Il n'y avait peu ou pas d'associations entre les marqueurs biochimiques (taux de CD4 et la charge virale) et les indicateurs de la qualité de vie liée à la santé. Toutefois, la qualité de vie a tendance à croître avec l'augmentation du taux de CD4. La faiblesse du non association entre les marqueurs biochimiques liés à la santé et les indicateurs de la qualité de vie liée à la santé montrent que ceux-ci peuvent ne pas être des mandataires directs les uns des autres et que la numération des CD4 et la charge virale peuvent être des critères inadéquats d'éligibilité à l'aide sociale.

## Introduction

Surrogate measures such as the CD4 count and viral burden have remained the major markers of disease progression and well-being of people living with HIV (PLWH). Such clinical indicators have also been deemed to provide an incomplete view of disease impact (Blaiss 1997; Kartz, Yelin, Eisner, Gillian & Blac 2004). To bridge this gap, health-related quality of life (HRQOL) measures are increasingly being incorporated in the traditional clinical measures of health. This shift is aimed at providing a greater depth of information on the impact of the disease on the physical, social and emotional well-being of individuals (Kartz *et al.* 2004). This kind of information is essential to ensure a higher efficiency, responsiveness and precision of the delivery of care and support services

HRQOL studies in HIV have mostly been cross-sectional in nature and focused mainly on issues such as functioning, adherence to treatment and perceptions about HRQOL (Hughes, Jelsma, Maclean, Darder & Tinise 2004; Louwagie, Bachmann, Meyer, Booysen, Fairall & Heunis 2007; Nicholas, Corless, Bhengu, Ncama, Mclnerney, McGibbon, *et al.* 2004; O'Keefe & Wood 1996; Phaladze, Human, Dlamini, Hulela & Mahlubi 1996). The use of cross-sectional designs contradicts the argument that authenticating the relationship between clinical measure of HIV and AIDS and HRQOL would require repeated measurements of the variables (Weinfurt, Willke, Glick, Friemuth & Schulman 2000). This assertion should also be read against the backdrop that the bio-psychosocial effects of HIV vary over time (Gurunathan, Habib, Baglyos, Meric, Plotkin, Dodet *et al.* 2009; Murdaugh 1998). The majority of studies available in South Africa have used generic quality of life assessment tools, which have their limitations, in measuring HRQOL in specific situations such as HIV and AIDS (McSweeny & Creer 1995). Among the limitations of generic tools are their inability to measure change following defined interventions over time and their failure to capture specific disease-related parameters. In view of this gap, the World Health Organisation developed a HRQOL instrument for PLWH (WHOQOL-HIV) (WHOQOL-HIV Group 2004).

Few studies have tried to link biomedical markers of the HIV and AIDS disease progression and HRQOL in developing counties in comparison to the number of such studies conducted in developed countries where the health and social support systems are well established. Given that the biomedical and HRQOL markers of HIV infected people also vary over time, there is also a limited record of studies that have examined how both measures change or interact over time. The WHOQOL-HIV instrument has never been tested over time among PLWH to assess its stability and applicability through the bio-psychosocial trajectory of HIV/AIDS. Against this background, this study sought to establish the relationship between the CD4 count, viral burden and HRQOL and their differences among treatment-naïve people with AIDS and people with HIV who have been on treatment for 12 months. The need for this study is heightened by the current dependence on the CD4 cell count and duration on treatment as the eligibility criteria for disability grants in South Africa.

## Methodology

This study is based on a secondary data analysis. The database consists of two cohorts made up of 311 treatment-naïve HIV positive people with AIDS defining characteristics and 331 HIV positive individuals who have been on treatment for 12 months. This resulted in a total of 642 records analysed in this study. This study is part of an original study that recruited participants from antiretroviral treatment clinics based in public hospitals. Convenient sampling of participants was used. The adoption of convenient sampling method is due to the absence of a sampling frame to be used for probability sampling methods. The clinics do not have a waiting list for treatment initiation as patients are placed on treatment as soon as they meet the requirement for antiretroviral therapy (ART) treatment initiation. As such, patients who were ready to commence treatment based on the treatment initiation criteria and who may have gone through the treatment readiness assessment and training processes were co-opted into the study.

Persons aged 18 years and above living with HIV and AIDS and accessing care in public health facilities were targeted. The treatment-naïve cohort was limited to persons presenting symptoms attributable to or due to complications from HIV infection and being initiated to treatment for the first time and people who have been on treatment for 12 months.

HRQOL was measured using the WHOQOL-HIV instrument. The WHOQOL-HIV is made up of 120 questions divided into 6 domains, namely: the physical; psychological; level of dependence; social relationships; environment and spirituality. The health and demographic data, participants’ CD4 counts and viral load were also part of the instrument. The CD4 count and viral loads were collected from patients’ medical records from routinely conducted tests. The instruments were administered in the respective local language of each participant.

The study data were managed using the Statistical Package for Social Sciences for windows version 18. Basic descriptive analysis of the participants' socio-demographic and quality of life measures was done and compared between the two cohort groups. This was followed by internal consistency assessment using Cronbach's alpha to assess the reliability of the instruments. Student *t*-tests and analysis of variance were used to compare the average HRQOL subscale and domain scores between times, key demographic characteristics and the biological markers. To maximize the data of the two cohorts in the study, combined/aggregate analysis of all the cases was also performed. The combined analysis was to augment the results of the changes observed between the two cohorts. Ethical approval for the study was granted by the University of the Witwatersrand Human Research Ethics Committee. The data used for the secondary analysis did not have any personal detail of the study participants and the analyses and the use of data in this study remained cognizant of issues of confidentiality and anonymity.

## Result

There were 642 cases in the study consisting of 311 treatment-naïve AIDS patients and 331 people with HIV who have been on treatment for a period of 12 months. The majority of the participants, in both groups, were women and people over 36 years of age, married, with children, unemployed and with only primary education. The proportion of the participants who belongs to support group was more than twofold higher among the 12 months treatment cohort when compared to the proportion in a support group among the treatment-naïve AIDS patients. The average age of the treatment-naïve participants was 39.3 years (standard deviation = 9.8), while the average age of participants in 12 months treatment cohort was 40.8 years (standard deviation = 9.7). The average monthly household income of the treatment-naïve was ZAR565.98 (standard deviation = 375.84) and the average monthly household income for the 12 months treatment cohort is R1490.37 (standard deviation = 984.77). Further description of these variables can be found in Table 1.

**Table 1.** Socio-demographic characteristics of study participants.

	Treatment-naïve cohort *n* (%)	12 months treatment cohort *n* (%)
Sex		
Male	127 (40.8)	117 (35.3)
Female	184 (59.2)	214 (64.7)
*Age group (years)*		
18–25	23 (7.4)	13 (3.9)
26–35	99 (31.8)	96 (29.0)
36–45	108 (34.7)	126 (38.1)
46+	80 (25.8)	96 (29.0)
*Marital status*		
Married	129 (42.9)	137 (41.5)
Never married	103 (34.2)	101 (30.6)
Divorced/separated/widowed	69 (22.9)	92 (22.9)
*Children*		
Yes	251 (84.2)	231 (71.7)
No	47 (15.8)	91 (28.3)
*Education*		
No education	51 (18.5)	142 (48.6)
Primary	134 (48.6)	102 (34.9)
Matric	73 (26.4)	45 (15.4)
Tertiary	18 (6.5)	3 (1.0)
*Employment*		
Yes	35 (11.3)	33 (10.2)
No	275 (88.7)	289 (89.8)
*Support group*		
Yes	42 (22.0)	179 (56.6)
No	149 (78.0)	137 (43.4)

The study noted higher CD4 cell count among the 12 months cohort and this is in comparison to the CD4 cell count among the treatment-naïve group. All the treatment-naïve participants had CD4 cell counts below 500 cells/μl, while 15% of the participant in the 12 months treatment cohort had greater than 500 cells/μl. While almost half of the 12 months treatment cohort had CD4 cell count of greater than 350 cells/μl, less than 1% of the treatment-naïve cohort had similar CD4 cell counts (*χ*^2^ = 326.94; *p* = .000; *n* = 623). Similarly, the proportion of people with suppressed viral load below 400 copies/ml was more among the 12 months treatment cohort (78.3%) when compared to the 16.3% viral load suppression below 400 copies/ml noted among the treatment-naïve group (χ^2^ = 75.64; *p* = .000; *n* = 339).

## HRQOL measures

Table 2 presents the average HRQOL scores among the two groups.

**Table 2.** Comparison of the average WHOQOL-HIV domain scores.

Domain	Cohort	*n*	Mean	SD	*p*-Value
Physical	Treatment-naïve	289	9.90	2.806	.000
	12 months on treatment	311	12.13	3.260	
Psychological	Treatment-naïve	281	11.88	2.484	.000
	12 months on treatment	308	13.61	2.031	
Level of independence	Treatment-naïve	284	9.20	2.515	.000
	12 months on treatment	313	11.52	2.519	
Social relationships	Treatment-naïve	283	11.89	1.850	.000
	12 months on treatment	312	13.21	2.543	
Environment	Treatment-naïve	266	11.32	1.769	.000
	12 months on treatment	311	13.49	1.681	
SRPB	Treatment-naïve	150	9.35	2.859	.000
	12 months on treatment	230	13.63	3.281	

The mean scores for all quality of life domains differed significantly between the two groups. Both groups recorded the lowest mean scores under the level of dependence domain. The treatment-naïve group recorded their highest quality of life score under the psychological domain, while the 12 months treatment cohort's highest score was under the Spirituality/Religion/Personal Beliefs (SRPB) domain.

For a better clinical and statistical judgment, the viral load and CD4 cell count were grouped into categories. The viral load was classed as suppressed and unsuppressed using 400 copies/ml as a cut-off point (Department of Health, 2003). The CD4 cell counts were, however, grouped into four (0–200 cell/μl; 201–349 cell/μl; 350–500 cell/μl; >500 cell/μl) (Peltzer & Phaswana-Mafuya 2008).

The correlation of these categories of biological markers with WHOQOL-HIV domains showed only two notably low associations between the viral load and level of dependence and environmental domains, respectively, among the treatment-naïve group. All the other correlations showed no association.

Table 3 illustrates the aggregate analysis correlating the biomedical markers with HRQOL indicators for all the participants combined.

**Table 3.** Combined correlation coefficients of the CD4 cell count level, viral suppressions status and the WHO–QOL domains of both cohorts.

	CD4 cell count grouping:			Viral load suppression status		
	*N*	*R*	Sig.	*N*	*R*	Sig.
Physical	581	.26*	.00	318	−.02	.73
Psychological	571	.32*	.00	313	.00	.94
Level of independence	579	.32*	.00	319	−.10	.07
Social relationships	578	.19*	.00	320	.00	.90
Environment	559	.38*	.00	315	.01	.86
SRPB	374	.36*	.00	220	−.07	.28

Note: SRPB: Spirituality/Religion/Personal Beliefs.

This table shows a slightly different pattern with CD4 cell count categories tending to correlate more with WHOQOL domains. The highest correlation coefficient observed was for the environment (*r* = .38, *p* = .000), SRPB (*r* = .36, *p* = .000) and psychological domains (*r* = .32, *p* = .000). Despite this, all the correlations were too low to suggest any association (Sim & Wright 2000).

Table 4 illustrates the average domain scores by the viral load suppression status among the two groups.

**Table 4.** WHOQOL-HIV domains scores by viral load suppression status.

	Treatment-naïve cohort			12 months treatment cohort		
	*N*	Mean	SD	*n*	Mean	SD
*Physical domain*						
Suppressed	7	11.71	4.15	212	12.39	3.37
Unsuppressed	41	9.41	4.14	58	11.52	3.18
*Psychological domain*						
Suppressed	5	12.64	2.22	215	13.87	1.97
Unsuppressed	39	12.02	3.05	54	13.10	1.91
*Level of independence domain*						
Suppressed	7	10.14	2.27	218	11.68	2.64
Unsuppressed	39	7.67	3.33	55	11.05	2.31
*Social relationships domain*						
Suppressed	7	11.61	2.45	215	13.25	2.63
Unsuppressed	40	13.07	2.00	58	12.93	2.53
*Environment domain*						
Suppressed	5	9.70	1.82	213	13.54	1.69
Unsuppressed	38	12.43	2.03	59	13.58	1.41

Note: Suppressed viral load was below 400 copies/ml and unsuppressed above 400 copies/ml.

Table 4 shows that the quality of life was better among the 12 months treatment cohort. In other words, the health quality of life scores for the 12 months treatment cohort were consistently higher than the quality of life of the treatment-naïve group with suppressed and unsuppressed viral loads. On face value, people with suppressed viral load had a higher quality of life at 12 months. Despite this, significant differences in the quality of life score between people with suppressed and unsuppressed viral loads was noted in only two instances. People with suppressed viral load had higher environmental domain score among the treatment-naïve cohort, while a significantly higher psychological domain score was noted among people with suppressed viral load in the 12 months treatment cohort. The highest domain score was also noted among people with suppressed viral load under the psychological domain at 12 months. On the other hand, people with unsuppressed viral load reported the lowest average quality of life domain score under the level of dependence among the treatment-naïve cohort.

Table 5 shows results of the aggregate analysis of the average quality of life scores by the viral load suppression status.

The combined analysis in Table 5 shows that the domain mean scores of the suppressed and unsuppressed participants differ significantly in three out of the six domains. The three domains were the physical, psychological and level of dependence. With the exception of the SRPB domain, participants with suppressed viral loads had higher quality of life scores in the remaining five domains. The mean scores on the level of dependence were relatively lower than the mean scores in the other five domains (Table 5). The highest mean scores reported were for the SRPB domain.

Table 6 compares the average domain scores by CD4 cell count categories across the two cohort groups.

**Table 5.** Comparison of the combined average WHOQOL-HIV domains cores by the viral load suppression status.

	Status	*n*	Mean	SD	*T*	Sig.
Physical	Suppressed	219	12.31	3.20	1.93	.00
	Unsuppressed	99	11.73	3.57	1.87	
Psychological	Suppressed	220	13.69	1.95	3.25	.00
	Unsuppressed	93	13.05	2.38	3.05	
Level of dependence	Suppressed	225	11.56	2.45	3.51	.00
	Unsuppressed	94	10.71	2.99	3.30	
Social relationships	Suppressed	222	13.25	2.55	−.22	.83
	Unsuppressed	98	13.30	2.27	−.22	
Environment	Suppressed	218	13.35	1.67	.55	.58
	Unsuppressed	97	13.26	1.70	.55	
SRPB	Suppressed	174	13.72	3.20	−.93	.35
	Unsuppressed	46	14.04	3.28	−.92	

Note: Suppressed viral load was below 400 copies/ml and unsuppressed above 400 copies/ml.

**Table 6.** WHOQOL-HIV domains scores by the CD4 count level.

	Treatment-naïve cohort					12 months treatment cohort				
	*N*	Mean	SD	*F*	Sig.	*n*	Mean	SD	*F*	Sig.
*Physical domain (CD4 cell counts in cells/µl)*										
0–200	254	9.95	2.87	.65	.52	58	12.34	3.24	2.53	.06
201–349	23	9.43	2.40			97	12.53	3.54		
350–500	2	11.50	4.95			101	11.52	2.99		
>500						46	12.89	3.07		
*Psychological domain*										
0–200	247	11.92	2.53	.14	.86	55	13.57	2.54	3.81	.01
201–349	23	12.17	1.94			98	13.53	1.91		
350–500	2	12.40	2.82			100	13.44	1.82		
>500						46	14.57	1.75		
*Level of independence domain*										
0–200	251	9.21	2.55	.05	.94	56	11.77	2.85	2.54	.06
201–349	22	9.05	2.17			99	11.71	2.69		
350–500	2	9.50	3.54			102	11.04	2.22		
>500						47	12.15	2.38		
*Social relationships domain*										
0–200	251	11.89	1.86	.30	.74	55	13.55	2.50	3.44	.01
201–349	22	12.15	1.50			101	13.54	2.70		
350–500	2	12.50	2.48			102	12.61	2.45		
>500						45	13.74	2.35		
*Environment domain*										
0–200	234	11.37	1.80	.21	.81	57	13.45	1.76	.87	.46
201–349	21	11.60	1.05			97	13.43	1.60		
350–500	2	11.00	3.54			101	13.54	1.67		
>500						47	13.88	1.65		
*SRPB domain*										
0–200	129	9.45	2.90	2.27	.13	38	14.34	3.50	5.37	.00
201–349	17	8.35	2.15			74	14.22	3.22		
350–500						86	12.58	2.98		
>500						30	14.53	3.26		

Table 6 shows that significant differences in the mean scores of the various categories were observed only among the 12 months treatment cohort in the psychological, social relationship and the SRPB domains. In the three domains, people with CD4 cell counts of over 500 cell/μl consistently had higher mean domain scores. The highest score was also reported under the psychological domain among the 12 months treatment cohort, while the lowest score was reported under the level of dependence domain among the treatment-naïve cohort (9.05).

A combined analysis of CD4 cell count categories by HRQOL domain indicate that the average scores reported under each of the CD4 cell count categories tended to be higher in the psychology domain and lowest in the level of dependence domain. The lowest mean scores were under CD4 cell count of 0–200 cell/μl in the level of dependence (10.17) and physical (10.95) domains. On the other hand, the highest mean scores were noted under CD4 cell count of >500 cell/µl under the psychology (14.63) and SRPB domains (14.86).

A post hoc test of the aggregate analysis showed the differences in the mean scores within the respective domains of the CD4 cell counts categories. The quality of life score tended to increase with increases in the CD4 cell count category. The differences between consecutive categories were mostly significant.

## Discussion

The objective of this study was to establish the relationship between the CD4 count, viral burden and HRQOL and their difference among treatment-naïve people with AIDS and people with HIV who have been on treatment for 12 months. With this, the study showed few weak correlations between CD4 counts and the quality of life measures. Similarly, the viral load suppression status had very low correlation coefficients with the overall quality of life. Another study conducted in South Africa reported similar correlations between quality of life domains and CD4 cell count (Venter, Gerike & Bekker 2009).

In line with this study's findings, Jia, Uphold, Zheng, Wu, Chen, Findley *et al.* (2007) have found a significant positive association between the CD4 cell count and emotional status of PLWH and people living with AIDS. This may explain the observed positive progression in the CD4 cell count and mean psychological domain score. The reported correlations were, however, too low for quality of life measures to become predictors of the CD4 cell count and viral load. This observation may be linked to the highly subjective nature of the quality of life measure and the many socio-cultural and demographic factors that influence their variation. Such variations may be profound even in the most homogenous populations given the expected differences based on age, sex, religious belief, ethnic group marital status, economic standing and the extent of social support and access to treatment and other health service.

Factors similar to the above are known to also affect an individual's rate of progression from HIV to AIDS (Geskus, Meyer, Hubert, Schuitemaker, Berkhout, Rouzioux, *et al.* 2005; Rangsin, Chiu, Khamboonruang, Sirisopana, Eiumtrakul & Briwn 2004; Touloumi, Pantazis, Babiker, Walker, Katsarou, Karafoulidou *et al.* 2004). In the same context, this and other studies have shown marked differences based on these variables (Peltzer & Phaswana-Mafuya 2008; Preez & Peltzer 2009; WHOQOL-HIV Group 2004). The study by Venter *et al.* (2009), for instance, showed marked differences in correlation coefficient across the various quality of life domains and CD4 cell counts by simply splitting their study population by sex.

This study also noted marked differences in quality of life between both cohort groups. This finding agrees with studies that have shown such improvements in the perceived quality of life over time (Solomon, Batavia, Venkatesh, Brown, Verma, Cecelia *et al*. 2009). This observation highlights the benefits of treatment in improving the health and HRQOL of PLWH and people living with AIDS. The progressively low scores in the level of dependence even at 12 months of treatment remains a major source of concern. This domain covers issues relating to mobility, activity of living tolerance, dependence on medication and capacity to work. This may again suggest that improvements in biomedical markers do not translate into individuals' ability to earn a living through work or caring for themselves. This point challenges exclusive reliance on the CD4 cell count and viral load as the eligibility criteria for accessing disability grants for PLWH.

The generally improved HRQOL over time noted by this study regardless of changes in the biomedical measure of disease progression may also be attributed to the natural psychosocial trajectory of HIV/AIDS prompted by treatment initiation and better psychosocial support. This assertion is premised on the model of achieving balance in the event of HIV infection and moves the individual across four stages – of disintegration, renormalizing, coming-to-terms and creating meaning – (Murdaugh 1998). Over time, this process creates a sense of control, predictability in the face of the many changes and a better understanding of the disease progression pattern. This occurs through exposure to varied psychosocial experiences and processes of adjustments to the disease manifestations from the point of diagnosis and through the respective stages of the disease. This is, however, not a linear process as various factors may positively or negatively alter the quality of life at different points in the HIV-illness trajectory. In the context of this study, treatment initiation results in improved health outcomes and sense of hope and potentially leads to better adjustment to the disease. This proposition also draws on the fact that quality of life improves with treatment initiation and availability of psychosocial support (Castro, Guerra & Casa de la Sal 1996; Mannheimer, Matts, Telzak, Chesney, Child, Wu *et al.* 2005). These two factors are the main differences between the two cohorts compared in this study.

In conclusion, this study has described the HRQOL of PLWH based on their position in their treatment history and noted the weak to no association between HRQOL indicators and the biomedical markers of HIV/AIDS such as the CD4 cell count and viral load. The study mostly underscores the multiple factors that affect the natural history of HIV/AIDS. Some of such factors are time, treatment and the socio-demographic characteristics of the individual. This proposition is also predicated on the observation that an individual's perceived quality of life and well-being is also defined by similar factors. With all the variations observed in this study, including the differences in the findings of the time specific and combined analyses, one can say that PLWH are highly heterogeneous in terms of clinical manifestations and support requirements and as such should only be grouped together with caution in research studies and intervention programmes. Furthermore, the general prominence of the psychological, environmental and level of dependence domains noted in this and related studies may require a further research to explore their link with the CD4 cell count and viral load.

A possible limitation of this study is that it is not a direct follow-up of the treatment-naïve AIDS patients to measure change over time, but it may serve as a good alternative in HIV and AIDS studies. This assertion is based on the expected bio-psychosocial trajectory of HIV and AIDS and the related differentials over time and along the natural history of the disease (Gurunathan *et al.* 2009; Murdaugh 1998).
